# Clinicopathological background of local recurrence in high grade sarcoma of the extremity with preoperative chemotherapy: a supplementary analysis of JCOG0304

**DOI:** 10.1093/jjco/hyaf027

**Published:** 2025-02-07

**Authors:** Satoshi Tsukushi, Kazuhiro Tanaka, Toshiyuki Kunisada, Ryunosuke Machida, Satoshi Takenaka, Akira Kawai, Hirohisa Katagiri, Masanobu Takeyama, Makoto Endo, Katsuhiro Hayashi, Robert Nakayama, Hiroshi Hatano, Makoto Emori, Shinichirou Yoshida, Toshio Kojima, Akio Sakamoto, Jungo Imanishi, Ryosuke Kita, Toshifumi Ozaki, Yukihide Iwamoto

**Affiliations:** Department of Orthopedic Surgery, Aichi Cancer Center, 1-1 Kanokoden, Chikusa-ku, Nagoya 464-8681, Japan; Department of Advanced Medical Sciences, Oita University, 1-1 Idaigaoka, Hasama, Yufu City, Oita 879-5593, Japan; Department of Medical Materials for Musculoskeletal Reconstruction, Okayama University Graduate School of Medicine Dentistry and Pharmaceutical Sciences, 2-5-1, Shikata-cho, Okayama, 700-8558, Japan; Japan Clinical Oncology Group Data Center/Operations Office, National Cancer Center Hospital, 5‐1‐1 Tsukiji, Chuo‐ku, Tokyo 104‐0045, Japan; Department of Orthopedic Surgery, Osaka International Cancer Institute, Osaka, Japan; Department of Musculoskeletal Oncology, National Cancer Center Hospital, 5‐1‐1 Tsukiji, Chuo‐ku, Tokyo 104‐0045, Japan; Division of Orthopaedic Oncology, Shizuoka Cancer Center, 1007 Shimonagakubo, Nagaizumi-cho, Sunto-gun, Shizuoka 411-8777, Japan; Department of Musculoskeletal Tumor Surgery, Kanagawa Cancer Center, 2-3-2, Asahiku, Yokohama, 241-8515, Japan; Department of Orthopaedic Surgery, Kyushu University, 3-1-1 Maidashi, Higashi-ku, Fukuoka, 812-8582, Japan; Department of Orthopaedic Surgery, Kanazawa University, 13-1 Takara-machi, Kanazawa, 920-8641, Japan; Department of Orthopaedic Surgery, Keio University School of Medicine Graduate School of Medicine, 35 Shinanomachi, Shinjuku, Tokyo 160-8582, Japan; Department of Orthopedic Oncology, Niigata Cancer Center Hospital, 2-15-3, Kawagishi-Cho, Chuo-Ku, Niigata 951-8556, Japan; Department of Orthopaedic Surgery, Sapporo Medical University, West 16, South 1, Chuo-Ku, Sapporo, 060-8543, Japan; Department of Orthopaedic Surgery, Tohoku University Graduate School of Medicine, 1-1 Seiryo-machi, Aoba-Ku, Sendai 980-8574, Japan; Department of Orthopaedic Surgery, Nihon University, 30-1, Oyaguchi Kami-Chou, Itabashi-Ku, Tokyo 173-8610, Japan; Department of Orthopaedic Surgery, Kyoto University, Shogoin, Kawahara-cho 54, Sakyo-ku, Kyoto 606-8507, Japan; Department of Orthopedic Surgery, Teikyo University, 2-11-1 Kaga, Itabashi-Ku, Tokyo 173-8606, Japan; Japan Clinical Oncology Group Data Center/Operations Office, National Cancer Center Hospital, 5‐1‐1 Tsukiji, Chuo‐ku, Tokyo 104‐0045, Japan; Department of Orthopaedic Surgery, Okayama University Graduate School of Medicine Dentistry and Pharmaceutical Sciences, 2-5-1, Shikata-cho, Okayama, 700-8558, Japan; Kyushu Rosai Hospital, 1-1 Sonekitamachi, Kokura Minami-Ku, Kitakyushu 800-0296, Japan

**Keywords:** soft tissue sarcoma, local recurrence, preoperative chemotherapy, surgical margin, adjuvant radiotherapy

## Abstract

**Background:**

The mainstay of treatment for soft-tissue sarcomas is complete resection with negative surgical margins. However, treatment strategies for local control including the frequency of adjuvant radiotherapy (RT) and surgical margin differ greatly between Japan and other countries, and the optimal strategy of local control remains controversial.

**Methods:**

A total of 70 patients with high-grade sarcoma who underwent surgery of the 72 patients enrolled in JCOG0304, were included. The primary endpoint was the proportion of local recurrence, and we investigated the clinicopathological background of local recurrence cases, including the surgical margins according to the Japanese Orthopedic Association (JOA) margin classification or histological margin, and use of adjuvant RT.

**Results:**

Local recurrence occurred in five patients, with a 5-year local recurrence proportion of 7.1% (95% confidence interval, 2.6%–14.8%) in 70 patients. The histological subtype were four cases of undifferentiated pleomorphic sarcoma (UPS) and 1 case of liposarcoma. The 5-year local recurrence proportions for UPS and non-UPS were 19.0% and 2.0%, respectively. Two of the five recurrent cases (40%) had adjuvant RT. The recurrent cases were four males and one female, median age 54 years (range: 33–66), JOA margin classification showed wide resection in four cases and marginal resection in one case, and histological margin showed negative in all five cases.

**Conclusion:**

Despite the low proportion of adjuvant RT, local control of high-grade soft tissue sarcoma with preoperative chemotherapy in JCOG0304 was good. However, more detailed surgical margin evaluation and the use of adjuvant RT should be further investigated in the future for UPS.

## Teaser

Local control of high-grade soft tissue sarcoma in JCOG0304 was good. Limb-salvage surgery in Japan based on the barrier concept for non-UPS might minimize the use of adjuvant radiotherapy.

## Introduction

Soft tissue sarcoma (STS) is a rare malignant tumor accounting for ⁓1% of all malignancies in the USA [1]. The mainstay of treatment for non-small STS is complete resection with negative surgical margins, and local recurrence is significantly associated with poor prognosis. The cumulative probability of local recurrence at 5 years reported in international large series ranges from 13% to 28% [2–6]. Various predictive factors, including age, tumor size, histological grade and surgical margin have been reported to significantly influence the risk of local recurrence [2–7].

Various systems exist for the classification of surgical margins. Before modern multiplane imaging, a system by Enneking *et al.* [8] for describing STS surgical margins described intralesional, marginal, wide, and radical margins, and noted improved local control through radical resections. Use of the American Joint Committee on Cancer (AJCC) residual tumor classification, R classification, is reported increasingly in sarcoma studies [9]. Several studies of surgical margins in extremity STS have used this definition and found it prognostic for local recurrence. On the other hand, Japan uses the Japanese Orthopedic Association (JOA) margin classification, a gross margin classification based on concept of barrier that reflect the resistance to tumor spread through structures such as thick fascia [10–12]. Few studies have compared the R classification with the JOA margin classification, and optimal margin classification for local control strategies is controversial. An ideal margin classification system would be prognostic for local recurrence and would be reproducible, to facilitate communication among clinicians and researchers.

Adjuvant radiotherapy (RT) for STS is widely used as the standard of care in the USA and Europe based on the evidence of a prospective randomized trial [13]. An analysis of the SEER database of 5075 cases who underwent surgery for truncal and extremity STS from 2004 to 2009 reported that 50% of all patients received adjuvant RT [14]. On the other hand, according to an analysis of the Japanese Bone and Soft Tissue Tumor Registry, there were 6344 limb-salvage surgeries for STS from 2006 to 2012, and adjuvant RT was limited to 979 cases (15%) [15]. In Japan, the goal is to achieve wide resection according to JOA margin classification, and RT is not used if adequate surgical margin is obtained. The use of adjuvant RT is limited to 13%–20% even in high-grade STS [16–18]. Therefore, treatment strategies for local control including the frequency of RT and surgical margin differ greatly between Japan and other countries [19,20], and the optimal strategy of local control remains controversial. However, there have been no previous detailed reports of local control in clinical trials using the identical treatment strategy.

We conducted a multicenter phase II trial, JCOG0304, that evaluate the efficacy of perioperative chemotherapy with doxorubicin (DOX) and ifosfamide (IFO) for treating high-risk STS of the extremities [21]. We have previously reported the perioperative chemotherapy with DOC and IFO was well tolerable and promising in the long-term results of JCOG0304 [16,22]. The aim of this study is to demonstrate the local recurrence-free probability and the clinicopathological background of local recurrence cases enrolled in JCOG0304.

## Patients and methods

All patient data used in this study were obtained from the patients who were enrolled in JCOG0304. The details of eligibility criteria of JCOG0304 have been published previously [16,22]. The major inclusion criteria of the trial were as follows: (i) A histological diagnosis of non-round cell STS including undifferentiated pleomorphic sarcoma (malignant fibrous histiocytoma), fibrosarcoma, leiomyosarcoma, synovial sarcoma, liposarcoma, pleomorphic rhabdomyosarcoma or undifferentiated sarcoma using open biopsy specimen; (ii) FNCLCC histological grading system: Grade 2 or 3; (iii) AJCC stage (the 6th edition): Stage III (T2bN0M0); (iv) resectable localized tumor in the extremities; (v) no history of treatment for non-round cell STS; (vi), no history of chemotherapy or radiation therapy for any cancer; (vii) age between 20 and 70 years; (viii) written informed consent. The patients were treated by preoperative chemotherapy with DOX (30 mg/m^2^/day, days 1 and 2) and IFO (2 g/m^2^/day, days 1–5), which was repeated three times every 3 weeks. Preoperative RT was not allowed in JCOG0304. The tumor was resected within 5 weeks from the last cycle of preoperative chemotherapy. Postoperatively, two cycles of DOX and IFO were carried out at intervals of 3 weeks.

The study protocol of JCOG0304 including secondary use of the trial data were approved by the Clinical Trial Review Committee of JCOG and by the Institutional Review Boards of each of the 27 participating institutes. JCOG0304 was conducted in accordance with the principles of the Helsinki Declaration and the Japanese Ethical Guidelines for Clinical Studies. Using data from all patients enrolled in JCOG0304, we performed supplementary analyses for local recurrence. Patients who underwent curative surgery in JCOG0304 were included in this supplementary study. The primary endpoint was the proportion of local recurrence, and the clinicopathological background including use of adjuvant RT, response of preoperative chemotherapy, and surgical margin classification (Histological and JOA) was investigated. The cumulative incidence of local recurrence was estimated by treating death before local recurrence as a competing risk. All statistical analyses were conducted using SAS 9.4.

### Evaluation of surgical margin

Margin status for each system was retrieved or determined from records. Histological margin classification: The margin was categorized as microscopically negative if there was no tumor at the surface, microscopically positive if tumor was at the surface. JOA margin classification [10–12]: The area of changed color, including hemorrhage and edema around the tumor capsule, was defined as the reactive zone. The margin was categorized either as wide if surgical margin includes the normal tissue outside the reactive zone, marginal if the margin is in the reactive zone, or intralesional if the margin passed through the tumor.

### Evaluation of response to preoperative chemotherapy

In JCOG0304, the response for the pre-operative chemotherapy was evaluated after the third course of chemotherapy using magnetic resonance imaging (MRI) according to modified Response Evaluation Criteria in Solid Tumor (RECIST) version 1.0 [16,23]. In this criteria, sum of the products of the maximum perpendicular diameters of lesions was calculated and the responses were classified into four groups; complete response (CR), no residual tumor; partial response (PR), 50% or greater decrease; stable disease (SD), no significant change (<50% decrease or < 44% increase); progressive disease (PD), 44% or greater increase. MRI of the tumor was obtained before enrollment of the patient for the baseline evaluation and after the pre-operative chemotherapy for the response evaluation. The response in all patients was also evaluated by the Central Response Review Committee of JCOG-BSTTSG to confirm the response of pre-operative chemotherapy for the tumor [16]. The pathological response to preoperative chemotherapy using the surgical specimen was also evaluated. The pathological response (tumor necrosis) was defined as follows: grade 0, necrosis ˂50% of the surgical specimen; grade 1, 50%–90% necrosis; grade 2, ˃90% necrosis; grade 3, no viable tumor cells.

## Results

**Figure 1:**
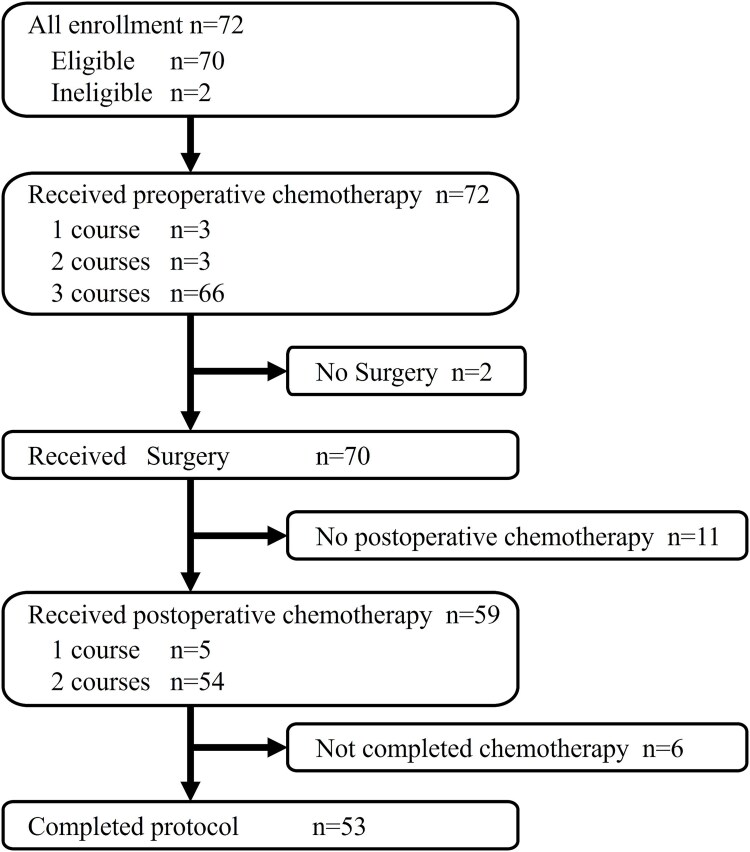
Study profile of JCOG0304. Among the 72 patients enrolled in JCOG0304, 70 patients who underwent curative surgery were included in this supplementary study.

From March 2004 to September 2008, 72 patients were enrolled into JCOG0304, and 70 of the 72 patients eligible for this supplementary study were included (Fig. 1). The characteristics of the included patients are summarized in Table 1. Briefly, 36 patients were males and 34 were females, and the median age of patients was 48.5 years old (range 21–66). The tumor location was the upper extremity in 14 patients, and the lower extremity in 56 patients. The median tumor size was 7.5 cm (3.0–26.0). The histological diagnosis of tumors was as follows: undifferentiated pleomorphic sarcoma (UPS) in 21 patients, synovial sarcoma in 19 patients, leiomyosarcoma in 12 patients, myxofibrosarcoma in eight patients, liposarcoma in six patients, and others in four patients. The JOA margin classification of the 70 patients was wide margin: 61 cases, marginal margin: eight cases, and intralesional margin: one case. The histological classification of the 70 patients was negative: 65 cases, and positive: five cases. Twelve patients (17%) had adjuvant RT. Pathological response to preoperative chemotherapy was grade 0 in 21 cases, grade 1 in 28 cases, grade 2 in 15 cases, grade 3 in five cases, and not evaluable in one case. Radiological response to preoperative chemotherapy was PR in 16 cases, SD in 51 cases, and PD in three cases.

**Table 1:** Clinicopathological data on 70 patients.

Characteristic	(N = 70)
Gender	
Male	36
female	34
Age (years)	
median	49
25%–75%	33–56
min–max	21–66
≥60	10
<60	60
PS	
0	51
1	19
Histological grade	
grade1	1
grade2	36
grade3	33
Site	
Upper extremity	14
Lower extremity	56
Histological subtype	
Undifferentiated pleomorphic sarcoma	21
Synovial sarcoma	19
Leiomyosarcoma	12
Myxofibrosarcoma	8
Liposarcoma	6
Others	4
Tumor size	
≦8 cm	38
>8 cm	32
JOA margin classification	
wide	61
marginal	8
intralesional	1
Histological margin classification	
negative	65
positive	5

Local recurrence occurred in five patients, with a 5-year local recurrence proportion of 7.1% [95% confidence interval (CI): 2.6%–14.8%]. The probability of local recurrence based on clinicopathological background are listed in Table 2. The 5-year local recurrence proportions for UPS and non-UPS were 19.0% (95% CI: 5.7%–38.4%) and 2.0% (95% CI: 0.2%–9.6%), respectively.

**Table 2:** The probability of local recurrence based on clinicopathological background.

	Characteristic	(N = 70)	No local recurrence	Local recurrence	LR (%)
Gender	Male	36	32	4	11
	female	34	33	1	3
Age	≥ 60	10	9	1	10
	< 60	60	56	4	7
PS	0	51	48	3	6
	1	19	17	2	11
Histological grade	grade1^a^	1	1	0	0
	grade2	36	34	2	6
	grade3	33	30	3	9
Site	Upper extremity	14	13	1	7
	Lower extremity	56	52	4	7
Histological subtype	UPS	21	17	4	19
	Synovial sarcoma	19	19	0	0
	Leiomyosarcoma	12	12	0	0
	Myxofibrosarcoma	8	8	0	0
	Liposarcoma	6	5	1	17
	Others	4	4	0	0
Tumor size	≦8 cm	38	36	2	5
	>8 cm	32	29	3	9
JOA margin classification	wide	61	57	4	7
	marginal	8	7	1	13
	intralesional	1	1	0	0
Histological margin classification	negative	65	60	5	8
	positive	5	5	0	0
	NE	0	–	–	–
Adjuvant radiation	Yes	12	10	2	17
	No	58	55	3	5
CT response (WHO criteria)	CR	0	–	–	–
	PR	16	15	1	6
	SD	51	47	4	8
	PD	3	3	0	0
CT response (pathological)	Grade0	21	21	0	0
	Grade1	28	26	2	7
	Grade2	15	14	1	7
	Grade3	5	4	1	20
	NE	1	0	1	100

LR, the probability of local recurrence (not considering competing risk). CT, chemotherapy; NE, not evaluable.

^a^One case enrolled as high-grade and assigned grade 1 by our central review was included in this study.

The characteristics of the recurrent cases are listed in Table 3. Four patients were males and one was a female, and the median age of patients was 52 years old (range 33–66). The tumor location was the upper extremity in 1 patient, and the lower extremity in four patients. The median tumor size was 7.5 cm (3.0–26.0). The histological diagnosis of tumors was as follows: UPS in four patients, and liposarcoma in one patient. JOA margin classification showed wide resection in four cases and marginal resection in one case, and histological margin classification showed negative in all five cases. Pathological response to preoperative chemotherapy was grade 1 in two cases, grade 2 in one case, grade 3 in one case, and not evaluable in one case. Radiological response to preoperative chemotherapy was PR in one case SD in four cases. Two patients (40%) had adjuvant RT.

**Table 3:** The characteristics of the recurrent cases.

Age	Gender	Site	Histology	Size (cm)	JOA margin	Histological margin	RT	CT response
66	Female	Lower leg	UPS	5.1	wide	negative	60 Gy	grade3
57	Male	thigh	UPS	14.5	wide	negative	no	grade1
52	Male	Upper arm	UPS	3.7	wide	negative	no	grade1
54	Male	buttock	UPS	11.2	marginal	negative	61.6 Gy	NE
33	Male	thigh	liposarcoma	12.0	wide	negative	no	grade2

NE, not evaluable.

## Discussion

The mainstay of treatment for STS is complete resection with negative surgical margins, and local recurrence is significantly associated with poor prognosis. In this study, we performed supplementary analyses for local recurrence using data from 70 patients enrolled in JCOG0304. Local recurrence occurred in five patients, with a 5-year local recurrence proportion of 7.1% in 70 patients, indicating good local control in the identical treatment strategy using preoperative chemotherapy (Table 4).

**Table 4:** The cumulative probability of local recurrence in international large series.

Study	Publication year	No. of patients	Adjuvant RT (%)	Positive margin (%)	Local recurrence (%)
Coindre JM [2]	1996	546	64%	29% (poor surgery)	28%
Gronchi A [3]	2005	911	37%	18%	19%
Weitz J [4]	2003	1261	44%	17%	21%
Zagars GK [5]	2003	848	95%	19%	21%
Gronchi A [6]	2012	314	93%	9%	13%
Current study	–	70	17%	7%	7%

RT, radiation therapy.

Treatment strategies for local control of STS including the frequency of adjuvant RT and the extent of surgical margin differ greatly between Japan and other countries, and the optimal strategy of local control remains controversial. Adjuvant RT for STSs is widely used as the standard of care in the United States and Europe based on the evidence of a prospective randomized trial [13]. The European Society of Medical Oncology (ESMO) clinical practice guidelines recommend wide excision followed by RT as the standard treatment for high-grade, deep lesions, >5 cm [20]. In Japan, however, adjuvant RT is considered a selective approach for inadequate margins due to concerns about long-term adverse events after high-dose irradiation and the high local control proportion achieved by surgery alone with adequate wide resection based on the JOA margin classification. The JOA practice guidelines state that there is no established consensus on which patients should receive adjuvant RT and that careful consideration must be given to the circumstances of each case when reviewing adjuvant RT [19]. Major centers in Japan have adopted a local control strategy based on the JOA margin classification using the concept of barrier that reflect the resistance to tumor spread through structures such as thick fascia. In the JOA margin classification, 2–3 cm of normal tissue without barriers or barriers such as fascia or periosteum is set as the surgical margin. In this study, only 7% of patients had positive margins, and a limited 17% of patients received postoperative adjuvant RT, reflecting this difference in treatment strategy.

There is no doubt that perioperative RT has a clear role in the local control of STS. However, adjuvant RT indeed can be detrimental, and the risk of early and late complications including delayed wound healing, flap necrosis, neuropraxia, joint stiffness, lymphedema, fractures and radiation-induced malignancy is widely recognized [24–26]. On the other hand, Gronchi *et al.* reported relatively good local control in a randomized clinical trial of perioperative chemotherapy in high-risk STS, with a 9% positive margin rate and a 13% local recurrence rate [6]. Although a high 93% of patients in this study were treated with adjuvant RT, the quality of limb salvage surgery might have an impact on local control. Over the past decade, advances in limb salvage procedures, including flap transfer and vascular reconstruction, have achieved adequate wide margins and improved local control of high-grade sarcomas at high-volume centers. This background has provoked debate about which patients can avoid adjuvant RT without increasing the risk of local recurrence. Several previous retrospective analyses and randomized trials have already suggested that where optimal margins can be achieved, RT has no additive effect on local control [27–31]. The JCOG-affiliated institutions in this study adopted a local control strategy based on the JOA system and achieved adequate wide margins. The 5-year local recurrence proportions for non-UPS were 2.0%. Although this study did not provide a definitive answer, limb salvage surgery based on the JOA system for non-UPS may minimize the use of adjuvant RT. The potential benefits of perioperative RT should be investigated, and its highly selective use considered in the future.

It is noteworthy that four of the five cases of local recurrence in this study were of the UPS histology. The 5-year local recurrence proportions for UPS were 19.0%, which was not sufficient local control. In the recurrence cases, JOA margin classification showed wide resection in four cases and marginal resection in one case, and histological margin classification showed negative in all five cases; therefore, three of the five patients did not receive adjuvant RT. This suggests that wider surgical margins are required to reduce the incidence of local recurrence in the UPS subtype known to have an infiltrative growth pattern. Fujiwara *et al.* investigated the effect of adjuvant RT for infiltrative subtypes of STS on the risk of local recurrence and observed a trend toward improved cumulative probability of local recurrence in patients with resected margins ˂10 mm, although the difference did not reach statistical significance [32]. For UPS, the definitive use of postoperative adjuvant RT based on more detailed surgical margin evaluation should be further investigated in the future for UPS.

In addition, local recurrence was observed in one of four (20%) grade 3 (total necrosis) pathological response to preoperative chemotherapy. Tanaka *et al.* suggested in the JCOG0304 trial that the death of sarcoma cells by preoperative chemotherapy does not always lead to the size reduction of STS [33]. High pathological response to preoperative chemotherapy might not have the benefit of limb salvage surgery in STS.

This study has some limitations. First, due to the limited number of cases of local recurrence, we were unable to evaluate multiple risk factors simultaneously. Despite this limiation, our study is valuable because there are few reports on local control in prospective clinical trials in which the treatment strategy is identical. To our knowledge, this is the first report of local control in a cohort of high-grade STS for which JOA and histological margin information has been prospectively collected. Second, this is an *ad-hoc* study based on data collected from prospective clinical trials. Therefore, detailed data on the width of surgical margins are not available. Previous retrospective studies have reported that the width of surgical margins of malignant STS has a significant impact on local control [34–36]. If investigated by such additional data in future prospective clinical studies, the optimal width of surgical margin to avoid adjuvant therapy might be validated.

## Conclusion

Despite the low proportion of adjuvant RT, local control of high-grade STS with preoperative chemotherapy in JCOG0304 was good. Although no definitive conclusions can be drawn from this study, limb-salvage surgery in Japan based on the barrier concept for non-UPS might minimize the use of adjuvant RT. However, more detailed surgical margin evaluation and the definitive use of adjuvant RT should be further investigated in the future for UPS.

## Notes

Trial registration: UMIN Clinical Trials Registry C000000096. Registered 30 August 2005 (retrospectively registered).
